# Cerebral blood flow and energy demand: imaging insights into neurovascular function

**DOI:** 10.1117/1.NPh.12.S2.S22810

**Published:** 2025-12-02

**Authors:** Alyssa Larios, Shivang Sullere, Chenghua Gu

**Affiliations:** Howard Hughes Medical Institute, Harvard Medical School, Department of Neurobiology, Boston, Massachusetts, United States

**Keywords:** neurovascular coupling, imaging, energy supply, cerebral blood flow

## Abstract

The brain depends on highly regulated moment-to-moment changes in regional blood supply to support its energetically demanding cognitive function with a limited energy budget. To efficiently match energetic supply to demand, neural activity rapidly increases regional blood flow. This process, known as neurovascular coupling (NVC), represents a particularly sophisticated form of functional hyperemia in the central nervous system, distinguished by its exceptional spatial precision. This feature of the cerebral vasculature generates a spatial and temporal relationship between neuronal activity and vasomotion. Although NVC is widely accepted to be essential for normal brain function and health, it remains poorly understood how NVC supports neuronal function and cognition. This review describes the current understanding of molecular and cellular mechanisms underlying NVC. We will also discuss the potential physiological functions of neurovascular coupling in normal brain function with a focus on energy supply to neural cells. Finally, the impact of neurovascular dysregulation on neurological disorders and the future outlook will be discussed.

## 1 Introduction

The brain accounts for only 2% of body weight, yet it consumes roughly 20% of the total energy budget with   70% to 80% of this energy consumption dedicated to maintaining resting membrane potentials and vesicular cycling.^1^^,^^2^ Although total brain perfusion is homeostatically held constant,^3^^,^^4^ the neurovascular network dynamically modulates regional blood volume to concentrate energy-rich blood toward highly active brain areas. This activity-dependent focal increase in cerebral blood flow, called neurovascular coupling (NVC), occurs when activated neurons trigger the dilation of nearby arterioles, leading to a localized increase in perfusion. Oxygen, once released from its chaperone hemoglobin, can freely diffuse through the vessel wall and cellular membranes to fuel mitochondrial respiration. Glucose, the most abundant carbohydrate in circulation, is transported from the vessels and into parenchymal cells via specialized transporters including glucose transporter 1 (GLUT1), GLUT3, and GLUT4.^5^

Most neurocognitive disorders present with concomitant cerebrovascular dysregulation,^6^^–^^8^ leading to the widespread appreciation that NVC is correlated with cognitive function. It has long been observed that blood flow in the cortex is markedly reduced in patients with mild to severe cognitive impairment, and this deficit often predates dementia or the detection of neurodegeneration.^9^^–^^11^ Moreover, decreased cortical blood velocity has even been used as a diagnostic tool for early Alzheimer’s disease (AD).^12^ In recent years, meta-analyses of AD progression with respect to biomarker severity have shown that vascular impairment might be the earliest pathogenic factor contributing to disease development.^13^ In addition, cerebral blood flow velocity is negatively correlated with amyloid accumulation in both human patients and mouse models for AD,^9^^,^^14^^,^^15^ suggesting a potential relationship where vascular dysfunction may be associated with amyloid deposition. Together, these reports show that impairment of NVC in the cortex is highly associated with neurological dysfunction and suggest that dysregulated vasomotion might play a causal role in disease progression or cognitive decline. This review aims to summarize the advancements in neurovascular coupling. We will also discuss the potential functions of neurovascular coupling in normal brain function, especially from the energy supply perspective. Finally, we will discuss the relationship between neurovascular dysregulation and neurological disorders as well as the outlook for future studies.

## 2 Organization of the Cerebral Vasculature

**Fig. 1:**
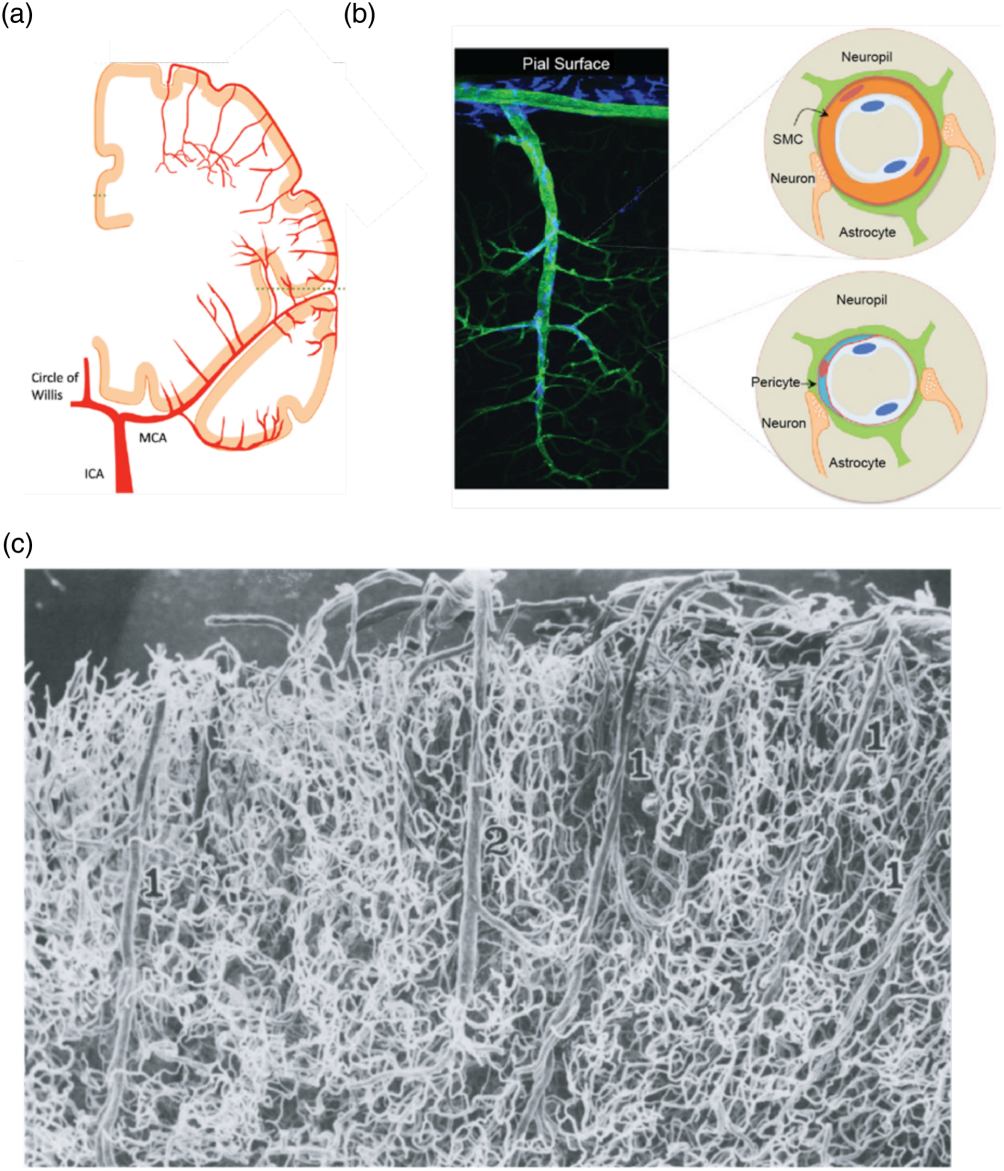
Anatomy of the cerebral vasculature. (a) Coronal illustration showing large ascending vessels and vascular organization on the brain’s surface. ICA, internal carotid artery. MCA, middle cerebral artery. (b) Histology showing a diving arteriole (green) and macrophages (blue), and a cartoon of vessel cross sections showing cellular components of a precapillary arteriole (top) and a capillary (bottom). Adapted from Iadecola.^6^ (c) Cast of human cerebral vasculature at 40× where a diving artery (1) and an ascending vein (2) is visible amongst the dense capillary bed. Adapted from Duvernoy et al.^17^

The anterior, middle, and posterior cerebral arteries emanate from the circle of Willis on the ventral surface of the brain and run parallel to the pial surface, bifurcating in a planar network. Arterioles then branch off, decrease in diameter, and dive down into the parenchyma. Smooth muscle cells (SMCs) encase arteries and arterioles by wrapping around the endothelial cell (EC) layer. SMCs are the effectors for blood flow modulation, as their relaxation dilates vessels and increases flux, whereas their contraction constricts vessels and decreases flux. Further downstream, arterioles become capillaries where SMCs transition into pericytes, mural cells that extend long processes axially along the abluminal face of the tubular ECs.^16^ Astrocytes tile the entire mural cell surface area with interdigitated endfeet on both capillaries and intraparenchymal arterioles [Figs. 1(a) and 1(b)].^6^ Cerebral capillaries arborize to such a degree that the mean distance between neuronal somata and the nearest microvessel measures 15 microns in the mouse cortex.^18^ The complexity of the cerebral capillary bed can be appreciated via casts of human vasculature,^17^ where the capillary network nearly covers the entire field of view compared with the sparser large vessels [Fig. 1(c)]. Recent advances in high-resolution 3D imaging have revolutionized our understanding of neurovascular organization. Serial two-photon tomography and tissue clearing techniques enable whole-brain mapping of the vasculature at single-cell resolution, revealing underappreciated heterogeneity in vascular density across brain regions.^19^^,^^20^ These imaging approaches have demonstrated that vascular architecture is precisely matched to regional metabolic demands, with particularly dense networks in metabolically active regions such as the hippocampus and sensory cortices.^21^

In rodents, the anatomical pattern of the anterior, middle, and posterior arteries is stereotyped, yet the patterning of the cerebral vasculature beyond these largest vessels is not consistent across individuals, indicating a degree of variability during development. Indeed, angiogenic sprouting is influenced by the extracellular environment in the early postnatal weeks; decreases in neural activity due to sensory deprivation or deafferentation lead to reduced vascular density. Likewise, increases in neural activity from sensory stimulation lead to increased vascular density.^22^ This supports the notion that angiogenesis matches the metabolic demands of the local environment. Interestingly, cortical vascular density correlates more with the number of synapses rather than the number of neurons in the human cerebrum,^17^ possibly because the relative energetic burden is skewed towards synapses. However, high-throughput histological reconstruction of the mouse barrel cortex angioarchitecture has demonstrated that vascularization density patterns across cortical layers are not as striking in rodents as they are in humans.^23^ Recent work using serial two-photon tomography to map cell types and cerebrovasculature at single-cell resolution in the whole adult mouse brain also revealed that neurovascular architecture meets regionally distinct brain energy regulation. Dense vascular networks were found in cortico-thalamo-striatal areas for motor-sensory processing. Quantitative analyses also revealed a positive correlation between vascular and capillary pericyte densities with parvalbumin^+^ (PV) interneurons and glutamatergic neurons.^19^

Although highly interconnected, the vascular network is structured into subdomains wherein diving arterioles perfuse their own columnar volumes of tissue. Targeted optical ablation leading to a single penetrating vessel occlusion results in a roughly cylindrical microinfarct.^23^^,^^24^ Functionally, two properties emerge from this organization. First, cortical tissue is particularly vulnerable to radial vessel blockages, as there is insufficient lateral flow between neighboring subdomains to compensate when a penetrating vessel is occluded. Second, blood flow can be targeted to specific cortical columns through changes in diving vessel diameter, allowing precise spatial control over perfusion domains.

## 3 Pathways for Cerebral Blood Flow Modulation

Although total cerebral perfusion is held constant, the brain harbors several pathways for global and local flow modulation. Systemically, the cerebrovascular tree responds to changes in blood gas concentration and arterial pressure on the order of seconds to globally regulate perfusion. Hypercapnia, or elevated ${\mathrm{pCO}}_{2}$ in the bloodstream, causes a strong vasodilation through direct action on SMCs via potassium and calcium channels.^25^^–^^27^ Slight deviations in arterial pressure within the physiological range of 60 to 150 mmHg in humans evoke autoregulatory SMC constriction and dilation to impede or facilitate flow, respectively, maintaining organ perfusion at an average 50  mL/100  g brain tissue/min.^3^ This myogenic response is reliant on mechanosensors in the vessel wall detecting hydrostatic and oncotic forces, the signaling cascades from which ultimately tune the SMC contractile apparatus through transient receptor potential (TRP) channel activation leading to SMC membrane depolarization and calcium influx.^28^^–^^31^

The brain’s intrinsic systems for flow modulation are twofold. The autonomic system appears to play a regulatory role over cerebral perfusion across varying brain states. Perivascular nerve terminals release vasoactive molecules such as acetylcholine, neuropeptide Y, and substance P directly onto vessel walls, inciting vasomotion on broad temporal and spatial scales. It is thought that autonomic innervation of the cerebrovascular system is involved in clinical indications such as migraine, but the pathways inducing the activation of perivascular nerves and their precise role in flow modulation remain unclear.^32^ Functional hyperemia, or NVC, is a more studied pathway for intrinsic cerebral blood flow control wherein neural activation leads to localized increases in perfusion. First described over one hundred years ago,^33^ neurovascular coupling has long been suspected to serve dynamic neuronal energetic demands by delivering commensurate levels of metabolic substrates.

### 3.1 Mechanisms Underlying NVC

Several mechanisms for neurovascular coupling have been proposed. Previous mechanistic models for NVC pathways implicate direct release of VIP onto arterioles by interneurons as a local integrator of neural activity into vasoactive signals, but these putative pathways involve autonomic afferents, which also release diffuse vasoactive agents onto the pial vasculature,^32^^,^^34^ limiting the regional specificity for evoked blood flow in these models. In fact, diving vessel dilation is spatially matched to neural activity such that arterial “receptive fields” in the visual cortex can be mapped to visual stimuli and are highly correlated with the orientation selectivity of the neurons within that perfusion domain.^35^

These observations raise the question: how does the vascular network detect regional changes in neural activity and transduce that signal into dilatory cues? The cellular pathways that mediate this signal transduction remain unclear; evidence suggests that multiple cell types may act in parallel to relay these signals to the effectors, SMCs. It has been posited that K+ released from active neurons opens inward-rectifier K+ (Kir2.1) channels on endothelial cells in the capillary bed, inciting a retrograde hyperpolarization propagated by gap junctions along the endothelium, which ultimately leads to smooth muscle relaxation in the feeding arterioles.^36^^,^^37^ Astrocytes, conveniently tethered to both synapses and vessels, have also been suggested to detect local neurotransmitter release and relay a signal in a calcium-dependent manner to the endothelium for upstream signal transmission.^38^^–^^40^ In addition, pericytes are positioned to sense extracellular cues to engage the endothelial signaling cascade, and several reports suggest that ATP-gated K+ channels on pericytes are activated during local neuronal activity, which is sufficient to produce upstream vasomotion.^41^^,^^42^ Finally, there is convincing evidence for direct neuron-to-SMC innervation, which could obviate the need for signal transduction involving other cell types. Synaptic-like glutamate transmission onto SMCs through N-methyl-d-aspartate (NMDA) receptors has been found to produce NVC responses,^43^ and neuropeptide Y (NPY) release from interneurons can produce vasoconstriction through the NPY-Y1 receptor on SMCs.^44^ In summary, nearly all cell types in the so-called “neurovascular unit” are capable of engaging vasodilatory signaling pathways. However, it is still unclear to what degree each of these pathways is activated in physiological conditions. Evidence from the past two decades has challenged the earlier view that metabolic byproducts initiate neurovascular coupling.^45^ Using combined functional ultrasound and two-photon imaging, researchers demonstrated that ${\mathrm{CO}}_{2}$ produced by neuronal metabolism does not contribute to activity-dependent vasodilation.^46^ These observations suggest that neurovascular coupling could operate through direct neural-vascular signaling pathways rather than metabolic intermediates, with response times of 0.5 to 1 s that precede any metabolic changes by several seconds.

Models with conduct electrical signals through the endothelium are attractive because it is consistent with electrical recordings done in peripheral skeletal muscle arteries, where the mechanisms for functional hyperemia were first explored.^47^^,^^48^ Second, the nearest vessels to the vast majority of neurons are capillaries whose capacity for active diameter control remains controversial.^49^^,^^50^ Although traditionally thought to lack contractile machinery, recent studies have provided conflicting evidence about whether capillary pericytes can actively modulate vessel diameter, with the debate often centering on the precise definition of capillaries versus pre-capillary arterioles. Thus, neuronal activity must be detected locally, and vasodilatory cues must be transmitted upstream for SMC relaxation. Moreover, severing just the endothelial cell layer in a pial vessel halts propagated dilation responses at the lesioned site during cortical stimulation, implicating endothelial cells as critical players for relaying neurovascular coupling signals along the vasculature.^51^ Recent study using visual and optogenetic stimulation paradigms to evoke spatially defined neural activity in awake mice while imaging the resulting vascular responses demonstrated that endothelial gap junctions enable long-range propagation of vasodilation signals through the vasculature during neurovascular coupling. Interestingly, the molecular composition of these gap junctions is zonated along the arterio-venous axis with arteries being the most strongly coupled segment. Acute genetic ablation of arterial endothelial gap junctions in adulthood results in blunting both the spatial extent and kinetics of vasodilation elicited by neural activity.^37^ Thus, the endothelium serves as a signaling highway for neurovascular coupling, allowing flexible and efficient distribution of limited energetic resources.

The precise pathways through which endothelial cells evoke smooth muscle relaxation are still under investigation. Nos3 encodes the endothelial nitric oxide synthase isoform (eNOS) and is constitutively expressed across all vessels. Its enzymatic activity for producing the potent vasodilator NO is induced by calcium influx or phosphorylation at one of several residues. To mediate smooth muscle relaxation, it is possible that hyperpolarization-induced signaling cascades in endothelial cells lead to eNOS catalysis via intracellular calcium influx or kinase activation. Two other isoforms, inducible and neuronal (iNOS and nNOS), have been detected at low levels in vascular endothelial cells, but their expression appears to be related to inflammatory or stress responses, and most data exploring their expression uses cell culture or dissociated cells, where transcription profiles may not reflect physiological levels.^52^

Post-translational modifications target eNOS to the cytoplasmic Golgi apparatus and plasmalemmal caveolae, which are caveolin-1 (Cav1)-coated pits in the plasma membrane.^53^ Cav1 is an integral membrane protein expressed in many different cell types across the body. It is known to interact with lipids and cholesterol, regulate membrane traffic through caveolae endocytosis, and act as a scaffold for signaling molecules.^54^ Interestingly, genetic deletion of arteriolar endothelial cell Cav1 leads to a 50% reduction of the neurovascular coupling response independent of eNOS signaling. An emergent model for relaying neural activity-evoked dilatory signals from endothelial cells to smooth muscle cells is that caveolae act to cluster and concentrate different ion channels for SMC relaxation and vasodilation.^55^ This study demonstrated that endothelial cells form a communication link between neural activity and SMC relaxation.

### 3.2 Functional Properties of NVC

Activity-induced increases in blood flow only emerge during the first few postnatal weeks. At P7, functional hyperemia is not observed in mice during sensory stimulation despite robust neuronal activation, and at P14, blood flow responses begin to emerge, albeit smaller in magnitude than in the adult. As a result, neuronal activation occurs in a local hypoxic environment.^56^ However, recent evidence suggests that aspects of NVC can be detected as early as P5 to P7, particularly when accounting for variations in arousal states.^57^ The time course of neurovascular coupling development appears to mirror that of vascular network maturation, suggesting that angiogenesis is elicited by hypoxic cues.^58^ In one hypothesis, when the brain is developing, the absence of neurovascular coupling reveals hypoxic loci representing regions with high levels of brain activity that would benefit from increased vascular density, inciting endothelial sprouting. Subsequently, as the vascular network matures, neurovascular coupling takes over as the primary means for increased local perfusion. This model suggests that functional measurements in younger subjects (neonates and infants) might display different cerebral blood flow patterns with respect to neural activity than in adults, requiring appropriate adjustments in blood-oxygen-level-dependent (BOLD) signal interpretations when analyzing functional magnetic resonance imaging (fMRI) data in humans for diagnostic purposes.

It has been widely suggested that the primary function of NVC is the delivery of blood-borne metabolic substrates to active neural populations. The brain has a remarkably low capacity for energy storage, and any existing stores tend to be exclusively found in astrocytes in the form of glycogen.^59^ Thus, neurons must continuously harvest fuel from the bloodstream to support general housekeeping and expensive signaling operations. Canonically, blood delivery is matched to neuronal activity for optimal metabolic support, but it has become clear that vasomotor responses and oxygenation changes do not strictly correspond to active neural territories nor are they proportional to activity. In fact, when receptive fields of diving arterioles in the visual cortex are mapped to visual stimuli, their response profiles are broader than the neurons in that perfusion domain.^35^ In other words, active neurons evoke increased blood flow in their own perfusion domain as well as other, less active domains. In addition, increases in tissue oxygenation substantially overshoot the cerebral metabolic rate of oxygen.^60^ This apparent oxygen oversupply may serve to maintain adequate oxygenation in regions farthest from capillaries, ensuring that even neurons at the periphery of vascular territories receive sufficient oxygen for oxidative metabolism.^61^ Other limiting substrates could include glucose or other nutrients. Indeed, neurons must uptake glucose during activation to maintain the vesicular cycle^62^, and some reports have suggested that glycolysis is the primary means for ATP generation in neurons during cortical activity.^63^ Still, the metabolic pathways fueling neuronal signaling are debated, and the extent to which neurons require increased oxygen or glucose delivery during activation is unknown.

### 3.3 Metabolic Pathways for ATP Generation

Because the brain has a very limited capacity for energy storage, it is critical that ATP is continuously generated. In the resting brain, it is observed that glucose and oxygen consumption are near the ideal stoichiometry for complete oxidation of glucose to carbon dioxide and water, indicating that glycolysis and oxidative phosphorylation are well-balanced. By contrast, acute neuronal stimulation leads to a transient increase in glucose consumption that is not accompanied by a proportional increase in oxygen consumption,^64^ indicating that the instantaneous energy demand of neuronal signaling may favor glycolytic pathways for ATP generation. This suggests that during activation, neural tissue leans on faster glycolytic pathways for ATP generation over slower but more fruitful oxidative pathways.

*In vitro* experiments showing that astrocytes can secrete lactate for neuronal uptake to fuel signaling are used as evidence for metabolic compartmentalization in the brain. Known as the astrocyte-to-neuron lactate shuttle (ANLS), this hypothesis suggests that astrocytes drive glycolysis and shuttle lactate to neurons where oxidative phosphorylation occurs.^65^^–^^67^ This model is controversial because little evidence supports that the ANLS occurs during brain activation—most experiments largely support the model’s feasibility.^68^ Supporting evidence includes observations that astrocytes preferentially express glycolytic enzymes and MCT1/4 lactate exporters, whereas neurons express MCT2 importers and oxidative enzymes, where systemic lactate injection results in a larger increase in lactate in astrocytes than in neurons.^69^ Furthermore, disruption of lactate transport can impair long-term potentiation and memory formation,^70^ suggesting potential functional importance.^71^ In direct opposition to the ANLS hypothesis, recent reports show that the glucose transporter GLUT4 is inserted into the presynaptic plasma membrane in neurons upon activation, and GLUT4 expression is required for normal presynaptic vesicular recycling.^62^ In addition, another report shows that neuron activation in the barrel cortex during sensory stimulation does not require lactate uptake.^63^ These studies support the notion that neurons rely on their own glycolytic machinery to synthesize ATP during activation. However, it is still possible that lactate shuttling from astrocytes to neurons occurs during resting states to fuel basal metabolism. It is also observed that both aerobic and anaerobic metabolism are upregulated during functional activation, indicating that no one pathway is fueling the brain’s energetic needs.^64^

The existing energy stores in the brain are almost exclusively in the form of astrocytic glycogen. It is debated whether astrocytic glycogen is broken down to fuel astrocytes themselves or neurons during increased activity, but some evidence suggests that increased glycogen content helps sustain neuronal firing in hypoglycemic conditions.^59^ Intriguingly, intrahippocampal injection of a glycogenolysis inhibitor in rats before an avoidance behavioral test disrupted long-term but not short-term memory,^70^ possibly implicating glycogen-derived lactate in the neuronal pathways involved in memory consolidation. Recent work shows that glycogen metabolism is regulated by noradrenergic signaling during arousal states, with β2-adrenergic receptor activation triggering glycogen breakdown that provides lactate specifically to active neural circuits.^72^ Beyond glucose, glycogen, and lactate, the brain can utilize alternative substrates under specific conditions. Ketone bodies can supply brain energy during prolonged fasting or a ketogenic diet, entering the tricarboxylic acid (TCA) cycle after conversion to acetyl-CoA. Indeed, brain ketone metabolism increases when blood ketone levels rise.^73^^,^^74^ Recent work demonstrates that astrocytes perform fatty acid β-oxidation not merely as emergency fuel but as a constitutive process required for normal cognitive function.^75^ Deletion of astrocytic CPT1A, the rate-limiting enzyme for fatty acid oxidation, impairs memory formation through disruption of the electron transport chain. Medium-chain fatty acids could also provide energy to neurons.^76^ These fatty acids bypass CPT1-mediated transport and directly enter mitochondria, yielding 4 to 6 ATP per carbon through β-oxidation. In addition, branched-chain amino acids could serve dual roles as nitrogen donors for neurotransmitter synthesis and replenishing the TCA cycle intermediate.^77^^,^^78^ Metabolic pathways and molecular players for ATP generation have been well reviewed in these articles.^79^^–^^85^

### 3.4 ATP Budgets

Theoretical estimations of ATP consumption in gray matter predict that the reestablishment of ionic gradients following action potentials and postsynaptic excitatory potentials accounts for nearly one-half and one-third of the total energetic expenditure in neurons, respectively.^2^ These values are derived from allocating ATP molecules to individual enzymatic reactions—such as Na+/K+ exchange across the membrane via a dedicated ATPase pump—and extrapolating how many reactions occur when a neuron fires. These calculations have been refined in more recent analyses, which account for measured physiological parameters in addition to theoretical predictions.^83^ However, empirical measurements show that action potentials consume significantly less energy than canonical budgets predict. In fact, the kinetics of voltage-gated sodium channels during an action potential allow sodium entry that is only 25% above the theoretical minimum, indicating that ionic flux is quite energetically efficient.^86^ Thus, ATP usage from Na+/K+ exchange after an action potential has been overestimated. By contrast, the cost of vesicular recycling at the synapse may have been underestimated, as acute blockage of ATP synthesis leads to a dramatic decrease in the endocytic efficiency at presynaptic terminals.^87^ Recent advances in synaptic bioenergetic studies reveal sophisticated metabolic adaptations. Presynaptic terminals maintain local glycolytic machinery, with activity-dependent recruitment of GLUT4 and glycolytic enzymes to support vesicle recycling independently of mitochondrial ATP production.^88^ The energetic cost of synaptic transmission scales nonlinearly with activity, as high-frequency stimulation (>10  Hz) triggers parallel metabolic programs.^89^ Supporting the notion that the largest energetic burden is synaptic in origin, simultaneous fMRI and neurophysiological measurements observe that BOLD responses are better predicted by field potentials than spiking behavior.^90^^,^^91^ Field potential recordings are summed electrical potentials that are influenced by both synapses and cell bodies in a volume. This leads to the supposition that increases in blood flow during cortical activation may primarily fuel the energetic demands associated with synaptic transmission. Given that the brain contains a multitude of cell types, it is likely that energetic demands vary dramatically across neuronal populations. PV-positive GABAergic interneurons are posited to consume a disproportionate amount of cerebral energy because of their fast-spiking nature and high probability of vesicular release. Consistent with this notion, ${\mathrm{PV}}^{+}$ interneurons are also enriched with mitochondria in all subcellular compartments compared to other cortical neurons,^92^ and their energetic efficiency per action potential is considerably lower than that of cortical pyramidal cells.^86^ They exhibit higher oxidative metabolism than pyramidal neurons, with enriched complex I and IV components that support their high firing rates. These cells also maintain higher mitochondrial density and threefold higher creatine kinase expression to buffer rapid ATP fluctuations.^92^^–^^94^ Thus, it has been suggested that this population is particularly vulnerable to metabolic stress, and perturbations in their signaling fidelity after hypoxic or hypoglycemic insult are the main contributors to pathological conditions such as epilepsy or cortical spreading depression. In hippocampal slice culture, there is some evidence that blockage of ATP synthesis affects fast-spiking cells differently than slow-spiking cells,^95^ but *in vivo* experiments are required to confirm whether these results apply to the intact physiological system.

### 3.5 Waste Clearance

Because neurovascular coupling is a conserved phenomenon across species, it is presumed necessary for normal brain activity, but its contribution to organ homeostasis is ambiguous. Many point to neurovascular coupling as a route for waste clearance. For example, vasomotion has been correlated with perivascular clearance of interstitial dye^96^ and subarachnoid pooling after hemorrhage.^28^ This idea has important implications in the context of aging when potentially neurotoxic deposits such as tau or amyloid-beta are considered: decreased clearing efficiency when vessels stiffen with age could facilitate the accumulation of extracellular aggregates associated with dementia. The extent to which vasomotion correlates with waste clearance and whether changes in vascular reactivity are associated with pathological accumulation of parenchymal solutes is unknown. There is some evidence that vasomotion correlates with fluid flow and waste clearance; one study found that transient irradiation of vessel walls in mice injected with intravenous fluorescent dextran led to paravascular dye clearance that was highly associated with vasomotion.^96^ Clearance was more rapid when nearby neurons were activated to induce local vasodilation. Another study found that amyloid clearance was accelerated by high bouts of neural activity and was washed out through paravascular flow.^97^ This suggests that solutes in the parenchyma can be mobilized through vascular-mediated fluid flow. In cases of impaired NVC, solutes may be less efficiently cleared and accumulate in the extracellular space. It would be an interesting line of study to investigate how increasing extracellular concentrations of neuronally derived molecules would affect excitability. Recent discoveries reveal that neuronal activity directly drives waste clearance through the glymphatic system. Neuronal activity and the associated K+ release trigger astrocytic endfoot Ca2+ signaling via inward-rectifier K+ channels, inducing pulsatile compression of perivascular spaces. This creates convective flow that clears metabolic waste including amyloid-β and tau. Initially, the clearance was thought to be most active during non-rapid eye movement (NREM) sleep when neuronal firing becomes highly synchronized, generating traveling waves of interstitial fluid flow that increase clearance rates.^14^^,^^96^^,^^98^^,^^99^ Recent work suggests that glymphatic clearance can also occur during wakefulness, particularly during specific brain states characterized by synchronized neural activity.^100^^–^^102^ The relationship between sleep states and clearance efficiency remains an active area of investigation.

In addition to the functions discussed above, potential alternative functions of NVC, such as supplying oxygen for neuromodulator synthesis, brain temperature regulation, signaling to neurons, and stabilizing and optimizing the cerebral vascular structure, can be found in this recent review.^103^

## 4 Cerebrovascular Dysregulation

Vascular dementia is the general moniker for cognitive decline that is associated with aberrant blood flow in the brain, and it has become apparent that AD and other neurodegenerative conditions often exhibit some form of vascular dementia, which contributes to disease progression. It is likely that clinical symptoms in these conditions are the result of a multitude of pathological pathways, a portion of which involve a vascular component, and these pathways together lead to neuronal dysfunction or death. The specific contribution of dysregulated cerebral perfusion to neuropathology and cognitive decline is under active investigation.

It has now been well-established that decreases in cerebrovascular reactivity and cerebral blood flow velocity often precede cognitive abnormalities in dementia. Critically, cerebral blood flow deficits and impaired neurovascular coupling responses may represent distinct pathophysiological processes. Although reduced baseline flow may result from cardiac and vascular malfunction, impaired NVC specifically reflects dysfunction in the mechanisms linking neural activity to vascular responses. A hallmark study surveyed cognitive performance, cerebral blood velocity, and hippocampal volumes over a 6.5-year period. They found that lower baseline blood velocity was predictive of dementia and hippocampal atrophy, and higher blood velocity correlated with larger hippocampal volumes.^11^ This study was perhaps the first to suggest that mild decreases in cerebral blood flow contribute to the development of dementia. Since then, other clinical factors affecting blood flow have been associated with cognitive deficits and neurodegeneration. Hypertension is hailed as a major risk factor for memory deficits, and atherosclerosis, or the thickening of arterioles, is predictive of developing dementia. Both hypertension and atherosclerosis are found to decrease the NVC response in animal models.^14^ Many other animal studies have observed vascular deficits, including impairments in NVC, preceding cognitive decline. For example, mouse models for AD overexpressing beta-amyloid show attenuated neurovascular coupling before amyloid-beta accumulation or cognitive deficits. Mice displaying pericyte degeneration, commonly seen in aging, show reduced NVC amplitude and delayed response kinetics months before cognitive impairment.^15^ Together, these observations in humans and animal models lead to the supposition that chronic neurovascular uncoupling due to some underlying vascular affliction plays a causal role in the development and progression of neuropathology.

One hypothesis is that vascular-derived changes in normal regulation of cerebral blood flow lead to a mismatch between energy supply and demand in active neural populations, increasing the chance of synaptic transmission failure. This may, in turn, decrease the drive from neurons to evoke vessel dilation, further dysregulating blood flow and affecting metabolic substrate delivery in a positive feedback loop. Although this is an appealing model to explain cognitive impairments associated with conditions affecting the vasculature, no study has rigorously evaluated the link between NVC and neuron excitability.

Several studies have attempted to directly investigate the repercussions of chronic cerebral hypoperfusion or neurovascular uncoupling, but the available tools have proven quite blunt. A common “mild hypoperfusion” model includes the complete ligation of one common carotid artery affecting cerebral blood flow. Unsurprisingly, it has been shown that this intervention leads to a host of pathological phenotypes including upregulation of inflammatory genes, accumulation of neurotoxic deposits, and behavioral abnormalities.^104^ Although these studies can reveal long-term effects of acute ischemia, they do not reveal the long-term effects of more subtle conditions dysregulating activity-driven cerebral blood flow. One study found that mice with ablated pericytes showed diminished neurovascular coupling and functional hyperemia, resulting in progressive cognitive decline over the course of several months.^15^ The authors interpret this finding as evidence that neurovascular uncoupling causes memory deficits, but pericytes are also known regulators in supporting the health of ECs. Thus, the progressive loss of pericytes could affect the integrity of capillaries, which could lead to neuropathology through a decrease in vascular density. Another study aiming to pharmacologically inhibit vasomotion administered a drug cocktail to mice, which reportedly blocked the hyperemic response to whisker stimulation. They found that after only 7 days of consistent drug administration, the animals performed significantly worse than control animals on learning and memory tasks.^105^ Although this is an intriguing result, systemic pharmacological agents could have unknown off-target effects in other tissues, complicating behavioral interpretation. To robustly investigate the effects of neurovascular uncoupling, a more targeted approach is necessary.

### 4.1 Imaging Neuronal, Glial, and Vascular Relationships

#### 4.1.1 Two-photon laser scanning microscopy

Two-photon microscopy is a central method for studying the neurovascular coupling at high spatial resolution. This approach enables imaging of fluorescently labeled neurons, glia, and microvessels at the pia and hundreds of microns below the cortical surface.^106^^,^^107^ The tight optical sectioning, availability of appropriate dyes and sensors, and multi-channel imaging approaches allow simultaneous monitoring of neuronal or astrocytic calcium activity along with quantifying changes in local capillary diameter and red blood cell (RBC) flux *in vivo*.^55^^,^^106^ Pioneering studies using two-photon laser scanning microscopy (2P-LSM) in rodents revealed fundamental aspects of neurovascular coupling, such as stimulus-evoked arteriole dilations (∼20% increases) concurrent with neuronal firing.^55^^,^^108^ By measuring blood flow speed and volume in individual microvessels concurrent with local cellular activity, 2P imaging has elucidated how neuronal activation triggers rapid focal hyperemia.^107^ Indeed, two-photon imaging has visualized how pericytes regulate local flow,^49^ investigates the role of astrocytes in dilations,^109^^–^^112^ and how these signals are propagated through larger networks.^37^ Such insights have been crucial in dissecting the roles of different cells in blood flow control.

#### 4.1.2 Three-photon microscopy

Three-photon excitation microscopy extends the reach of multiphoton imaging to deeper brain regions (∼>1  mm) by using longer infrared excitation (typically 1300/1700 nm).^106^ Using 3P, Texas Red-labeled vasculature and neurons can be imaged in CA1 hippocampus at ∼1.2 to 1.3 mm depth.^113^^,^^114^ Three-photon microscopy (3P) imaging in a mouse model carrying the Alzheimer’s risk gene Apoe4 revealed reduced baseline capillary flow in deep white matter and a blood flow drop potentially linked to white matter damage.^115^ Such findings underscore 3P microscopy’s power to probe blood perfusion in deeper brain regions.

#### 4.1.3 Functional magnetic resonance imaging

fMRI in rodents offers a noninvasive, whole-brain view of neurovascular function with increasing resolution. Using ultra-high magnetic fields (7 to 17 Tesla), small-bore MRI scanners can achieve voxel sizes on the order of 100×100×200  μm in mice.^116^ High-field fMRI also enables measurement of cerebral blood flow using approaches such as cerebral blood volume (CBV)-weighted imaging and arterial spin labeling (ASL).^117^

#### 4.1.4 Functional ultrasound imaging

Functional ultrasound imaging (fUS) is an emerging neuroimaging modality that harnesses high-frequency ultrasound to map brain activity via cerebral blood flow changes.^118^^,^^119^ The key advantage of fUS is its relatively large field of view. In mice and rats, fUS can cover an entire coronal brain section (∼1  cm depth and several mm width) with a pixel size ∼100  μm and achieve frame rates of 1 to 10 Hz for volumetric scans.^120^ Notably, by applying localization techniques (tracking intravenously injected microbubble contrast), ultrasound localization microscopy can further improve spatial resolution to the ∼10  μm scale, enabling mapping of brain-wide microvascular networks and their activity.^121^^,^^122^

#### 4.1.5 Laser speckle imaging

Laser speckle imaging (LSI) (also called laser speckle contrast imaging, LSCI) uses coherent laser light to illuminate tissue with a granular “speckle” interference pattern. Motion of RBCs causes fluctuations in the speckle pattern through which one can infer relative blood flow. This technique provides full-field, real-time perfusion maps without scanning, resulting in high spatiotemporal resolution.^123^^,^^124^ In this way, LSI enables measuring dynamic CBF changes in vivo using a simple, cost-effective, and robust method.^123^^,^^124^

#### 4.1.6 Intrinsic optical signal imaging

Intrinsic optical signal imaging (IOSI) relies on changes in cerebral blood volume and hemoglobin, oxy- and deoxyhemoglobin concentration shifts. IOSI measures “intrinsic” changes in reflectance of brain tissue to infer hemodynamics at high temporal resolution.^125^ IOSI has been used to chart retinotopic maps in visual cortex, tonotopic maps in auditory cortex, somatotopic maps in somatosensory areas, and olfactory maps^125^ by measuring hemodynamic signals triggered by specific stimuli. In the context of neurovascular coupling, IOSI provided early evidence for the tight link between neuronal activity and local blood flow/oxygenation changes.^126^^,^^127^ In addition, IOSI has been combined with fluorescent reporters in “wide-field optical mapping” setups—for instance, simultaneous imaging of intrinsic hemodynamics and GCaMP calcium signals or voltage indicators to directly relate neural activity to blood flow changes.^37^^,^^128^

## 5 Conclusion and Perspectives

*In vivo* imaging in awake mice technologies have significantly advanced our understanding of NVC mechanisms. However, the physiological functions of NVC for our brain are still not clear. In this review, we focused on the potential role of NVC in energy supply to neurons. Future studies should aim to answer the following key questions: Is NVC required for faithful neuronal signaling? Does NVC impairment affect neuronal activation or signal processing? Would chronic NVC impairment contribute to neuronal death or cognitive deterioration? Future work may further explore the mechanistic links between vascular dysfunction and sensory-evoked neuronal activity, highlighting the cerebral vasculature as a key regulator of normal circuit function.

## Data Availability

This paper does not present any original data. Any data cited would need to be provided by the original sources.
